# Sclerosing Mucoepidermoid Carcinoma With Eosinophilia of the Thyroid: A Rare Case From Georgia and a Comprehensive Literature Review

**DOI:** 10.7759/cureus.72739

**Published:** 2024-10-30

**Authors:** Giorgi Akhvlediani, Elene Dzodzuashvili, Nino Gabidzashvili, Bakur Arabuli, Keti Tsomaia, Mikheil Jangavadze

**Affiliations:** 1 Department of Endocrinology, American Hospital Tbilisi, Tbilisi, GEO; 2 Department of Internal Medicine, Tbilisi State Medical University, Tbilisi, GEO; 3 Department of General and Endocrine Surgery, American Hospital Tbilisi, Tbilisi, GEO; 4 Aleksandre Natishvili Institute of Morphology, Ivane Javakhishvili Tbilisi State University, Tbilisi, GEO; 5 Department of Pathology and Forensic Medicine, Faculty of Medicine, Ivane Javakhishvili Tbilisi State University, Tbilisi, GEO

**Keywords:** chronic lymphocytic thyroiditis, georgia, histopathology, sclerosing mucoepidermoid carcinoma with eosinophilia (smece), thyroid gland neoplasm

## Abstract

Sclerosing mucoepidermoid carcinoma with eosinophilia (SMECE) is a rare thyroid malignancy typically linked to chronic lymphocytic thyroiditis. We present the first documented case of SMECE in Georgia, involving a 41-year-old woman with Hashimoto’s thyroiditis. A 16 mm hypoechoic thyroid nodule was detected on routine ultrasound, and fine needle aspiration categorized it as Bethesda V. The patient underwent total thyroidectomy with central neck dissection, confirming SMECE confined to the thyroid without lymph node involvement (0/22 nodes). She recovered well postoperatively on hormone replacement therapy, with no recurrence during follow-up. This case emphasizes the importance of considering SMECE in patients with thyroid nodules, especially with autoimmune thyroiditis. Key diagnostic features include eosinophilic infiltration in a sclerotic stroma and mucinous and squamous differentiation. Immunohistochemistry, showing positivity for CK7, p63, and CEA, was critical for diagnosis. Given the absence of extrathyroidal spread, the patient did not receive radioactive iodine (RAI) therapy. This report adds to the limited literature, underscoring the need for careful histopathological and immunohistochemical evaluation in rare thyroid malignancies.

## Introduction

Sclerosing mucoepidermoid carcinoma with eosinophilia (SMECE) is a rare malignancy of the thyroid gland, first described by Chan et al. in 1991 [1]. This unique thyroid tumor is morphologically distinct from other thyroid and salivary gland-type neoplasms, presenting a diagnostic challenge due to its histological overlap with more common thyroid malignancies. SMECE is typically associated with chronic lymphocytic thyroiditis, an autoimmune disorder of the thyroid, and is often accompanied by marked eosinophilic infiltration and dense stromal sclerosis [2]. The tumor predominantly affects middle-aged females, with a significantly high female-to-male ratio, as reported in the literature [2,3].

Despite the growing recognition of SMECE, fewer than 60 cases have been documented globally, and there remains a limited understanding of its pathogenesis [4]. The tumor is characterized by slow growth and, in about half of the cases, demonstrates extrathyroidal extension with frequent involvement of regional lymph nodes [4,5]. Histopathologically, SMECE is marked by a mix of squamous and mucinous cells, set against a background of chronic inflammation. Fine needle aspiration cytology (FNAC) and imaging are typically inconclusive, often requiring surgical resection and thorough histopathological analysis for diagnosis [5].

We present the first documented case of SMECE in Georgia. This report aims to increase awareness of this rare tumor, outline its diagnostic challenges, and provide insights into its management.

## Case presentation

A 41-year-old woman, a lifelong non-smoker, presented to the outpatient clinic for a routine follow-up due to a history of Hashimoto’s thyroiditis diagnosed in 2020. The patient had been on thyroid hormone replacement therapy since diagnosis, with stable thyroid function tests. She reported mild fatigue and myalgia over the preceding months but denied any significant neck pain, dysphagia, or other systemic symptoms.

A routine neck ultrasound revealed a solid, hypoechoic thyroid nodule in the right lobe, with the largest dimensions measuring 16.3 x 14.6 mm, irregular margins, and both peripheral and weak central vascularization. Multiple small, hypoechoic lymph nodes in the central neck compartment (level VI) were identified, with the largest measuring 12 x 3.7 mm.

**Figure 1:**
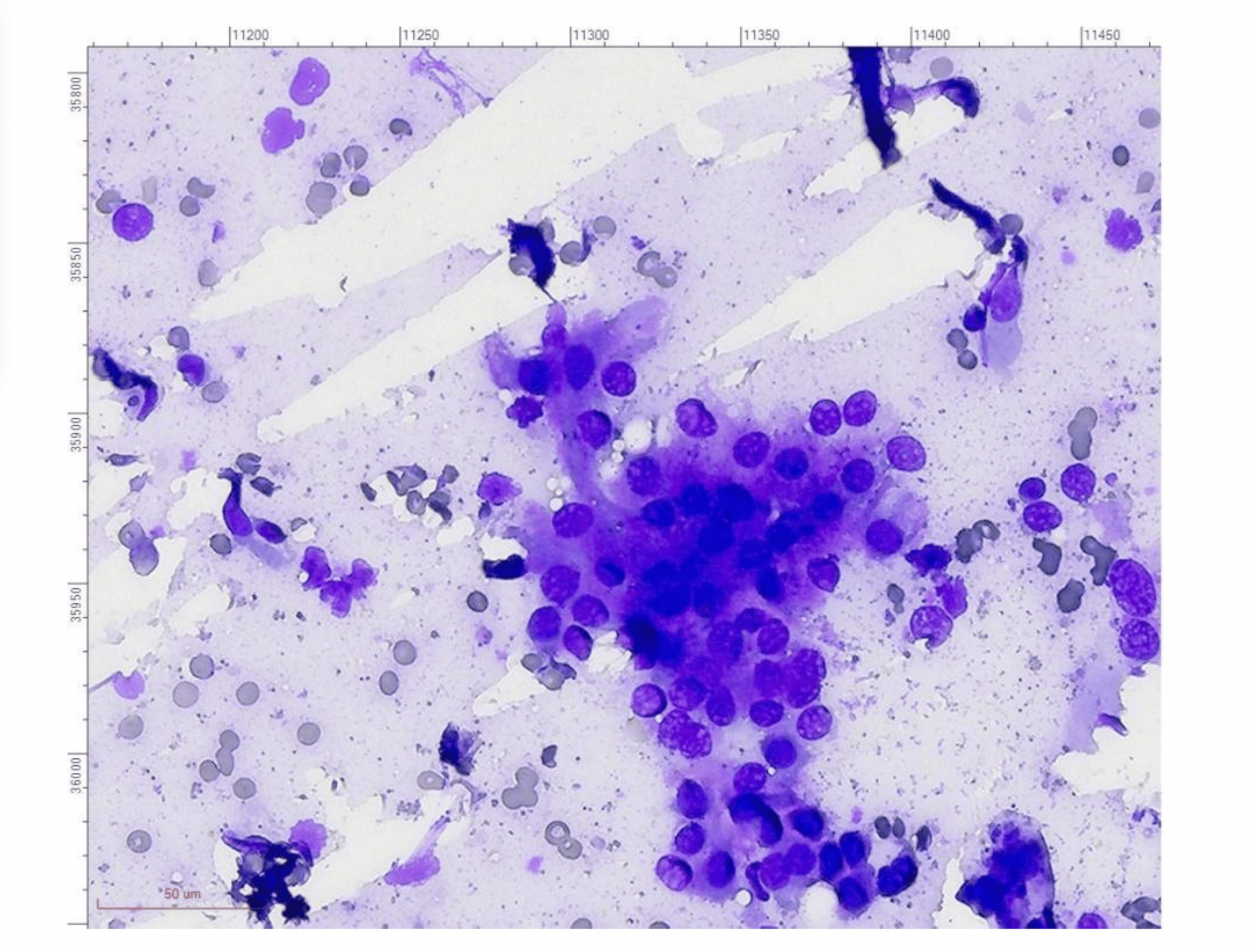
FNA cytology of the thyroid nodule (Giemsa stain) Scanned by MoticEasyScan Pro 6, Resolution 40X: 0.26 μm/pixel. Fine needle aspiration cytology demonstrated the presence of atypical cells, but no clear signs of malignancy were identified (a red line (marked in the figure) indicates a measurement of 50 μm).

FNAC was performed on the thyroid nodule and classified as Bethesda Category V, revealing atypical cells but without definitive malignant features (Figure 1). Serum calcitonin levels were normal, and serum thyrotropin (TSH) levels were within the reference range.

**Figure 2:**
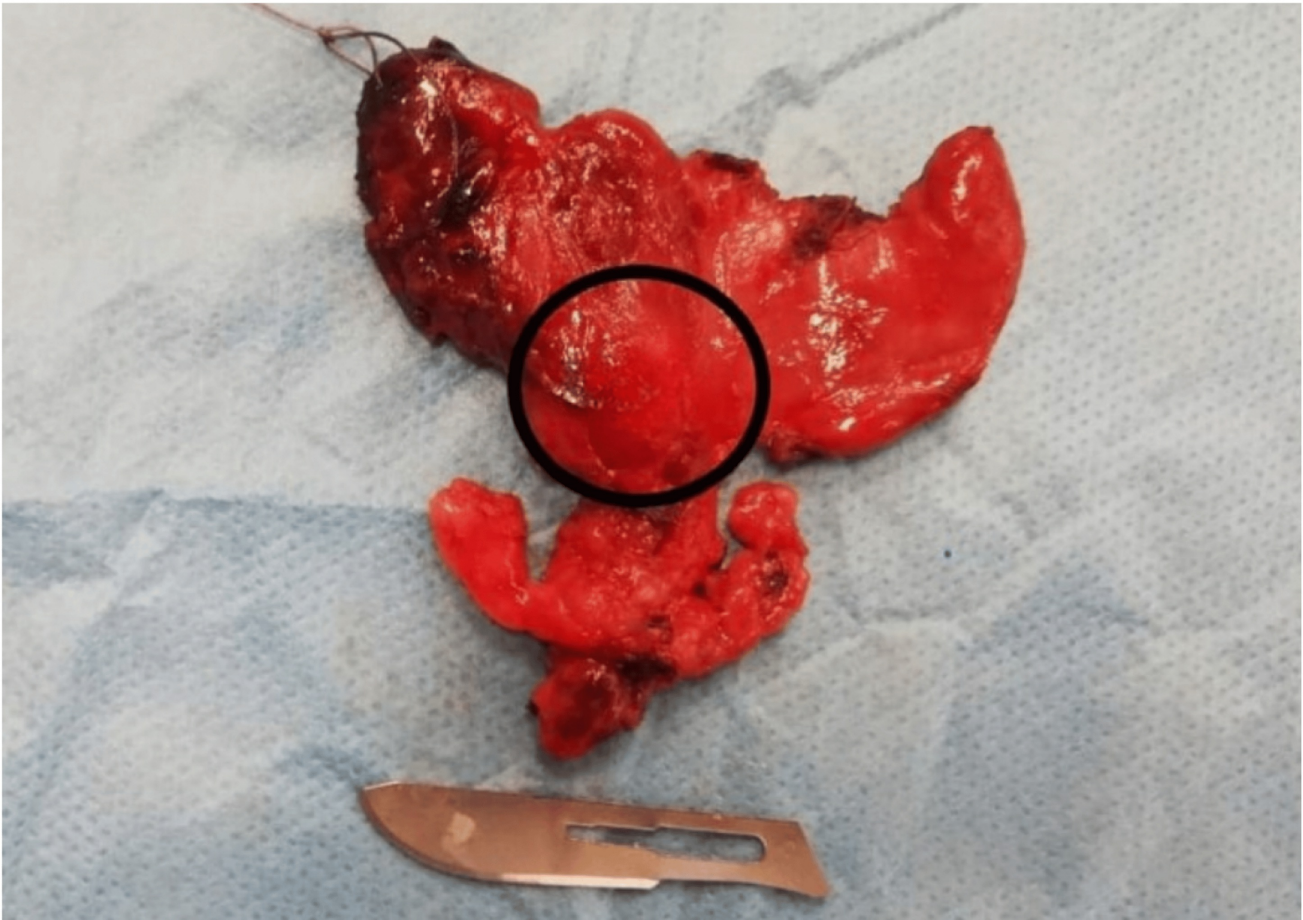
Gross examination of the thyroid specimen A solid nodule, measuring 15 mm, was grossly visible within the surgical specimen, located in the lower right lobe of the thyroid gland. A #10 blade scalpel (approximately 3.8 cm in length) is placed adjacent to the specimen for size comparison.

Due to the suspicious cytology, the patient underwent a total thyroidectomy with central compartment neck dissection. Intraoperative findings revealed a well-defined, firm mass within the right thyroid lobe, extending into the isthmus. Twenty-two lymph nodes were harvested from the central neck compartment (paratracheal and paraesophageal), all of which were negative for metastatic disease. The resected thyroid specimen measured 4.5 x 3.0 x 2.0 cm and contained a firm, well-circumscribed, solid nodule in the right lower lobe. The nodule was 15 mm in diameter, with ill-defined margins, and was white tan on the cut surface. No evidence of capsular invasion or gross extrathyroidal extension was observed (Figure 2). The surgical procedure was uncomplicated, with no intraoperative or major postoperative complications reported.

**Figure 3:**
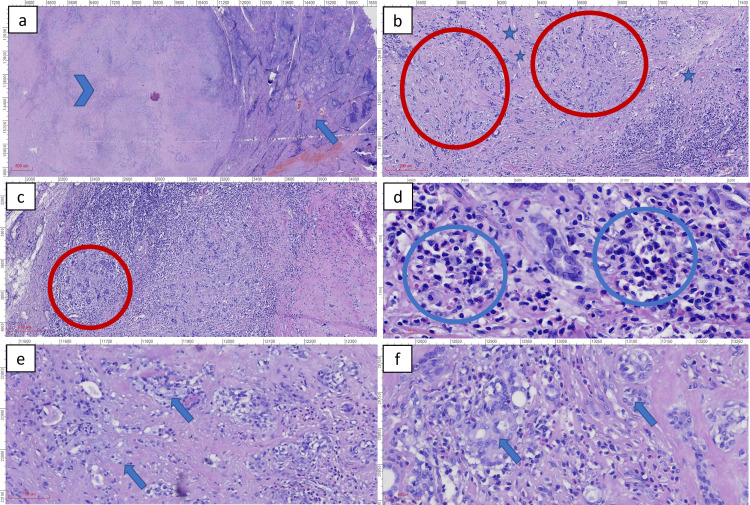
Histopathological features of sclerosing mucoepidermoid carcinoma with eosinophilia (SMECE) - hematoxylin and eosin (H&E) stain Scanned by MoticEasyScan Pro 6, Resolution 40X: 0.26 μm/pixel. (a) The tumor (arrowhead) is seen in the background of autoimmune thyroiditis (blue arrow) (a red line (marked in the figure) indicates a measurement of 800 μm). (b, c) Fibrosis or sclerosis of stroma (asterisk); tubular–glandular and cordal structures of tumor cells (red circle) (a red line (marked in the figure) indicates a measurement of 200 μm). (d) Abundant eosinophilic infiltrate throughout the tumor (blue circle) (a red line (marked in the figure) indicates a measurement of 50 μm). (e) Tumor cells exhibiting mucinous differentiation (arrow) (a red line (marked in the figure) indicates a measurement of 100 μm). (f) A nest of epidermoid component exhibiting squamous differentiation (arrow) (a red line (marked in the figure) indicates a measurement of 50 μm).

Microscopic examination revealed a heterogeneous tumor within a background of chronic autoimmune thyroiditis, characterized by lymphocytic infiltration and residual thyroid follicles (Figure 3a). The tumor exhibited extensive stromal fibrosis and sclerosis, with tubular-glandular and chordal structures (Figures 3b-3c). Tumor cells displayed both squamous (Figure 3f) and mucinous differentiation (Figure 3e), forming nests and cords of epithelial cells embedded within the sclerotic stroma. A notable feature was the presence of abundant eosinophils infiltrating the sclerotic stroma (Figure 3d), a hallmark of SMECE. Mitotic activity was rare, and there was no evidence of lymphovascular or perineural invasion. Importantly, all 22 lymph nodes were free of metastatic disease, and the surgical margins were clear.

**Figure 4:**
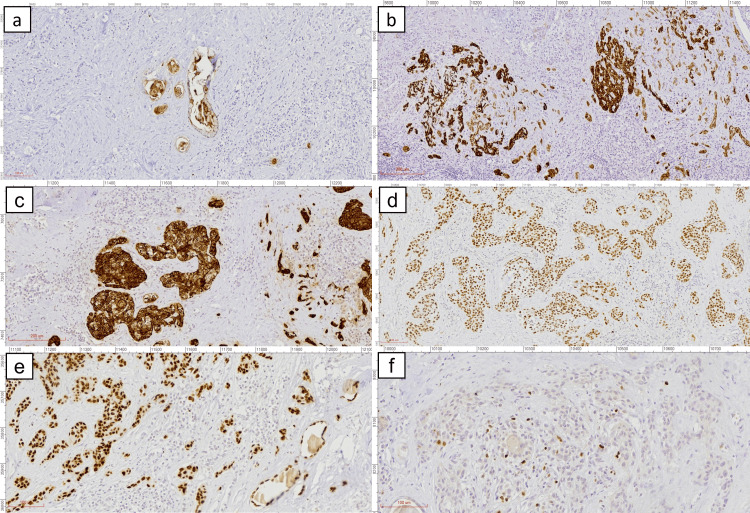
Immunohistochemical staining of SMECE tumor cells Scanned by MoticEasyScan Pro 6, Resolution 40X: 0.26 μm/pixel. a) CEA - positivity in Mucin-producing gland (a red line (marked in the figure) indicates a measurement of 100 μm). b) CK7 - positivity in Mucin-producing gland (a red line (marked in the figure) indicates a measurement of 200 μm). c) CK5 - positivity in squamous epithelial cells (a red line (marked in the figure) indicates a measurement of 200 μm). d) p63 - positivity in squamous epithelial cells (a red line (marked in the figure) indicates a measurement of 100 μm). e) TTF1 - positivity in tumour cells (a red line (marked in the figure) indicates a measurement of 100 μm). f) ki67 - nuclear positivity in tumor cells with approximately 5% nuclear positivity (a red line (marked in the figure) indicates a measurement of 100 μm).

Immunohistochemical analysis revealed strong positivity for CK7 in the mucinous tumor cells and p63 in the squamous component (Figures 4a-4b). The tumor cells also demonstrated reactivity for CEA (Figure 4c), and the squamous cells showed CK5 positivity (Figure 4d). TTF-1 expression was diffusely positive throughout the tumor (Figure 4e), consistent with thyroid origin. Ki-67 staining showed low proliferative activity, with approximately 5% of tumor nuclei displaying positivity (Figure 4f). Negative staining for calcitonin, synaptophysin, and CD56 ruled out medullary thyroid carcinoma. These findings confirmed the diagnosis of SMECE.

Following total thyroidectomy, the patient was initiated on a higher dose of thyroid hormone replacement therapy to maintain optimal thyroid function. During the immediate postoperative period, the patient developed transient hypoparathyroidism, which manifested as mild hypocalcemia. This condition was effectively managed with oral calcium supplementation and calcitriol. Serum calcium levels were monitored regularly, and supplementation was gradually tapered as the patient’s parathyroid function recovered.

Serum parathyroid hormone (PTH) levels were initially low postoperatively but returned to normal within four weeks, reflecting recovery of parathyroid function. The patient tolerated the increased thyroid hormone replacement dose well, with no signs of over- or under-replacement. Regular monitoring of thyroid function tests was conducted to ensure stable hormone levels (Table 1).

**Table 1:** Postoperative laboratory findings and progression This table outlines the key laboratory findings at different time points following surgery, including thyroid function, calcium levels, and parathyroid hormone (PTH) levels. It illustrates the patient’s biochemical response to treatment, showing the recovery of parathyroid function within four weeks postoperatively and stable thyroid hormone levels following an increase in thyroid hormone replacement therapy.

Test	Reference Range	Day 1 Post-op	2 Weeks Post-op	4 Weeks Post-op
Thyroid-Stimulating Hormone (TSH)	0.4 - 4.0 mU/L	0.9 mU/L	1.1 mU/L	0.8 mU/L
Free T4	0.8 - 1.8 ng/dL	1.4 ng/dL	1.5 ng/dL	1.6 ng/dL
Calcium (Ca)	8.5 - 10.2 mg/dL	7.8 mg/dL	8.6 mg/dL	9.1 mg/dL
Parathyroid Hormone (PTH)	15 - 65 pg/mL	8 pg/mL	32 pg/mL	48 pg/mL
Calcitriol	18 - 65 pg/mL	22 pg/mL	45 pg/mL	55 pg/mL
Thyroglobulin (Tg)	< 1.0 ng/mL (post-thyroidectomy)	0.2 ng/mL	0.1 ng/mL	< 0.1 ng/mL

Given the absence of extrathyroidal extension and the lack of lymph node involvement, radioactive iodine (RAI) therapy was deemed unnecessary. This decision was based on the favorable histopathological findings, which suggested a low risk of recurrence. The patient has been closely followed up with routine neck ultrasound and clinical examinations. At 12 months post-surgery, there have been no signs of disease recurrence, and the patient remains asymptomatic with stable thyroid function.

## Discussion

SMECE is an exceedingly rare thyroid malignancy, with fewer than 60 cases reported globally since its initial description by Chan et al. in 1991 [1,4]. SMECE presents a diagnostic challenge due to its histopathological overlap with more common thyroid carcinomas, such as papillary and medullary thyroid cancer, as well as salivary gland-type tumors [5]. Recent studies further emphasize the difficulties in differentiating SMECE from other thyroid malignancies due to these overlapping features [6]. This case, the first documented in Georgia, adds to the limited body of literature, demonstrating the complexity of diagnosing and managing this rare condition [7].

The patient, a 41-year-old woman, presented with a 16 mm hypoechoic thyroid nodule discovered during a routine follow-up for Hashimoto’s thyroiditis. Although FNAC is the standard initial investigation for thyroid nodules, in SMECE, it often yields inconclusive results, as seen in this case, where atypical cells were identified but without clear signs of malignancy. This challenge is consistent with findings in the literature, where SMECE is frequently misdiagnosed preoperatively due to the lack of definitive cytological features [7,8]. The co-occurrence of chronic lymphocytic thyroiditis in the majority of SMECE cases further complicates the diagnosis, as thyroiditis can mask or obscure malignant features [9].

Histopathologically, SMECE is characterized by a mix of squamous and mucinous cells set within a sclerotic stroma, heavily infiltrated with eosinophils [4]. These features were present in our patient, and immunohistochemical analysis, which showed CK7 and p63 positivity in the mucinous and squamous components, respectively, was crucial in differentiating SMECE from other thyroid malignancies. Diffuse TTF-1 positivity confirmed the tumor's thyroid origin. Similar findings have been consistently reported in other cases, where CK7 and p63 play a pivotal role in diagnosis, distinguishing SMECE from other tumors, such as anaplastic carcinoma or papillary carcinoma with squamous metaplasia [4,7,10,11].

The management of SMECE remains a subject of debate, given its rarity and the lack of standardized treatment guidelines [5]. Sciscent et al. also highlighted the variability in treatment approaches, particularly the role of total thyroidectomy and the decision to pursue RAI therapy [6]. In most cases reported in the literature, total thyroidectomy is the primary treatment, occasionally combined with central neck dissection, particularly when there is evidence of lymph node involvement. In this case, although the ultrasound revealed suspicious hypoechoic lymph nodes, none of the 22 dissected nodes showed metastatic involvement. Consequently, the decision was made not to pursue RAI therapy. The literature suggests that RAI is typically reserved for cases with extrathyroidal extension or nodal metastasis [4], both of which were absent in this patient.

The long-term prognosis for SMECE patients is generally favorable, as the tumor typically demonstrates a low mitotic rate and infrequent lymphovascular or perineural invasion [4]. However, cases of regional lymph node involvement and distant metastasis, including to the lungs and bones, have been documented, emphasizing the importance of careful postoperative monitoring [12]. In our case, the patient has been followed for 12 months postoperatively with no signs of recurrence, but long-term surveillance remains crucial. This aligns with reports from other cases, which have demonstrated the potential for late recurrence, sometimes several years after initial treatment [13]. The literature advises routine follow-up imaging to monitor for such recurrences [11].

Interestingly, the female predominance in SMECE, with a reported female-to-male ratio of approximately 16:1, is consistent with the epidemiology of other thyroid cancers [1,14]. The average age of diagnosis is typically in the mid-50s, though this patient, at 41, was slightly younger than average. This highlights the broad age range of patients with SMECE, as cases have been reported in individuals ranging from the second to the eighth decade of life [5]. Such variability in age and clinical presentation underscores the need for clinicians to maintain a high index of suspicion for SMECE, particularly in patients with a history of chronic thyroiditis and indeterminate cytology results [4,15].

## Conclusions

SMECE of the thyroid gland is a rare and challenging diagnosis that requires a combination of histopathological and immunohistochemical analysis for confirmation. Due to its rarity, there are no established treatment protocols, and management should be individualized based on tumor characteristics and clinical presentation. This case report highlights the need for awareness of SMECE among clinicians and pathologists, particularly in patients with thyroid nodules and underlying chronic lymphocytic thyroiditis. Long-term follow-up is crucial due to the potential for recurrence and metastasis, even in cases with favorable histological features. The scarcity of cases limits broader conclusions, and longer follow-up may be needed to fully assess recurrence or metastasis potential.

## References

[1] Chan JK, Albores-Saavedra J, Battifora H, Carcangiu ML, Rosai J (1991). Sclerosing mucoepidermoid thyroid carcinoma with eosinophilia. A distinctive low-grade malignancy arising from the metaplastic follicles of Hashimoto's thyroiditis. Am J Surg Pathol.

[2] Pantola C, Kala S, Athar M, Thakur S (2016). Sclerosing mucoepidermoid carcinoma with eosinophilia of the thyroid: a cytological dilemma. J Cytol.

[3] Heaven CL, Shetty S, De Beer JG (2022). Sclerosing mucoepidermoid carcinoma with eosinophilia of the thyroid gland: first Australasian case report. Ann Thyroid.

[4] Quiroga-Garza G, Lee JH, El-Naggar A (2015). Sclerosing mucoepidermoid carcinoma with eosinophilia of the thyroid: more aggressive than previously reported. Hum Pathol.

[5] Baloch ZW, Solomon AC, LiVolsi VA (2000). Primary mucoepidermoid carcinoma and sclerosing mucoepidermoid carcinoma with eosinophilia of the thyroid gland: a report of nine cases. Mod Pathol.

[6] Sciscent BY, Eberly HW, Bhele S, Goyal N (2024). Sclerosing mucoepidermoid carcinoma with eosinophilia: a diagnostic challenge. OTO Open.

[7] Iftikhar H, Awan MS, Ghaloo SK, Fatima S (2019). Sclerosing mucoepidermoid carcinoma with eosinophilia of thyroid. BMJ Case Rep.

[8] Bondeson L, Bondeson AG (1996). Cytologic features in fine-needle aspirates from a sclerosing mucoepidermoid thyroid carcinoma with eosinophilia. Diagn Cytopathol.

[9] Wenig BM, Adair CF, Heffess CS (1995). Primary mucoepidermoid carcinoma of the thyroid gland: a report of six cases and a review of the literature of a follicular epithelial-derived tumor. Hum Pathol.

[10] Hunt JL, LiVolsi VA, Barnes EL (2004). p63 expression in sclerosing mucoepidermoid carcinomas with eosinophilia arising in the thyroid. Mod Pathol.

[11] Lai CY, Chao TC, Lin JD, Hsueh C (2015). Sclerosing mucoepidermoid carcinoma with eosinophilia of thyroid gland in a male patient: a case report and literature review. Int J Clin Exp Pathol.

[12] Sukumar JS, Sukumar S, Purohit D, Welch BJ, Balani J, Yan S, Hathiramani SS (2019). Activating BRAF mutation in sclerosing mucoepidermoid carcinoma with eosinophilia of the thyroid gland: two case reports and review of the literature. J Med Case Rep.

[13] Le QV, Ngo DQ, Ngo QX (2019). Primary mucoepidermoid carcinoma of the thyroid: a report of a rare case with bone metastasis and review of the literature. Case Rep Oncol.

[14] World Health Organization (2017). WHO Classification of Tumours of Endocrine Organs: WHO Classification of Tumours, 4th Edition, Volume 10. WHO Classification of Tumours of Endocrine Organs: WHO Classification of Tumours, 4th Edition, Volume 10.

[15] Ames E, Campbell MJ, Afify A, Krane JF, Huang EC (2018). Sclerosing mucoepidermoid carcinoma with eosinophilia: cytologic characterization of a rare distinct entity in the thyroid. Diagn Cytopathol.

